# Symbols and grounding in large language models

**DOI:** 10.1098/rsta.2022.0041

**Published:** 2023-07-24

**Authors:** Ellie Pavlick

**Affiliations:** Department of Computer Science, Brown University, Providence, RI, USA

**Keywords:** natural language processing, cognitive science, language models

## Abstract

Large language models (LLMs) are one of the most impressive achievements of artificial intelligence in recent years. However, their relevance to the study of language more broadly remains unclear. This article considers the potential of LLMs to serve as models of language understanding in humans. While debate on this question typically centres around models’ performance on challenging language understanding tasks, this article argues that the answer depends on models’ underlying competence, and thus that the focus of the debate should be on empirical work which seeks to characterize the representations and processing algorithms that underlie model behaviour. From this perspective, the article offers counterarguments to two commonly cited reasons why LLMs cannot serve as plausible models of language in humans: their lack of symbolic structure and their lack of grounding. For each, a case is made that recent empirical trends undermine the common assumptions about LLMs, and thus that it is premature to draw conclusions about LLMs’ ability (or lack thereof) to offer insights on human language representation and understanding.

This article is part of a discussion meeting issue ‘Cognitive artificial intelligence’.

## 1. Introduction

Current work in artificial intelligence is dominated by the success of neural networks. The most visible success in recent years is that of large language models (LLMs), i.e. large neural networks which are trained on a word prediction task [1–4]. These models are particularly remarkable for their ability to generate fluent natural language text and dialogue at a level that is often indistinguishable from a human. However, despite many informal (and frequently overstated) claims about ‘human-like’ or ‘human-level’ language abilities [5], it remains unclear whether these impressive engineering achievements can offer insights to the study of language and cognition more broadly. The goal of this article is to present a case that they can: that is, that neural networks (LLMs specifically) can serve as plausible models of language understanding in humans.

I focus on two common criticisms of LLMs: (i) their lack of *symbolic structure* (§2), a phrase I use broadly to encompass a collection of criticisms including sample inefficiency, poor out of distribution generalization, lack of compositionality and inability to reason logically, and (ii) their lack of *grounding* (§3), i.e. the fact that they are trained only on text and thus have no access to or awareness of the physical, perceptual, goal-oriented or social contexts in which language occurs. Both of these criticisms are typically supported by empirical data demonstrating that LLMs perform extremely poorly on tasks that appear to require symbols [6–9] or grounding [10,11], respectively.

My counterargument is based on a premise that is commonplace in cognitive science: performance is not the same as competence, and analysing the performance of a system alone offers only limited insight into the system’s underlying competence.^1^ Until we can precisely characterize the representations and mechanisms in play under the hood, examples of LLMs’ behavioural successes or failures tell us little about LLMs’ ability to serve as models of language in humans. Of course, it can be argued that requiring analysis of the internal processing of LLMs amounts to holding LLMs to a higher bar than that to which we hold humans. We obviously cannot inspect humans’ internal neurological processing with the level of precision or invasiveness at which we can in principle inspect LLMs. It is true that this is a higher bar, and to a large extent, that is the entire point. If we want to consider LLMs, or any computational model, as a candidate model of the human mind, we must know something about how they work under the hood. Black box predictive models do little to advance understanding. Importantly, though, this higher bar holds whether we want to make positive or negative claims. Until we understand how LLMs work, we cannot assert that their internal processing bears any resemblance to humans, but we also cannot assert that it bears no resemblance. Undoubtedly, a precise characterization of neural networks’ representations and mechanisms is not trivial to acquire and will take time. But there is no rush. Work is already happening that brings us closer to characterizing this internal structure [12–14], and once such findings are mature, we can reanalyse this behavioural evidence and draw much stronger conclusions, positive or negative.

Thus, my goal in this article is not to argue *per se* in favour of one side in either the symbol or the grounding debate. Rather, the primary conclusion is that the debate about whether LLMs should influence how we think about human language processing is still ‘anybody’s game’. With this in mind, I intentionally focus on arguing for what, from my experience, is the minority opinion among those whose work is closest to these questions. That is, I argue (i) that neural networks can encode compositional symbolic structure without inductive biases (induced through training objective or architecture) towards such structure and (ii) that LLMs trained on text alone can capture the essential parts of linguistic meaning. In each case, I support the claim with a combination of in-principle arguments and empirical studies which inspect the internal structure of the representations and processes within the neural network.

Overall, I argue that it is premature to presuppose categories of problems that models like LLMs cannot or will not solve. Modern neural networks are in their infancy and our understanding of how they work is nascent. However, studies which seek to interpret the internal representations of neural networks have often revealed interpretable structures and processes, reminscient of existing theories of cognition. Thus, the question of whether LLMs can inform our theories of human language understanding is first and foremost an empirical question. And the next decade (hopefully much less) of work on understanding how neural networks work under the hood is likely to move the needle significantly.

### (a) Scope

I take the primary question at issue to be: what are the fundamental representations and processing algorithms that explain human language competence? Specifically, we are interested in the part of language which we call ‘meaning’ or ‘understanding’. There is little disagreement that LLMs can be (and indeed are) a good model of the shallower, predictive component of language processing [15], and even increasing agreement that LLMs may be good models of syntax and grammar [16]—once a very controversial claim [17]. Thus, the worthwhile debate necessarily concerns their ability to serve as models of meaning.

This article focuses on the potential of LLMs to serve as steady-state models of adult human language understanding. I do not claim that LLMs can serve as models of human language *learning*. On the surface, there is no debate to be had here. LLMs acquire their representations by reading the internet repeatedly and predicting each word in turn. At risk of being pedantic: children do not learn this way. An argument can (and has) been made that modern LLMs’ training mimics evolution, not development [18]. This is an interesting avenue to pursue, but to my knowledge, there is not yet any empirical work of relevance to the claim. Thus, while questions of learning are ultimately intimately intertwined with the questions at issue here [19], such questions are out of scope for this article primarily because there is not (yet) experimental evidence from artificial intelligence to inform an argument one way or the other.

This article is not a literature review. Other recent reviews have been published which are more comprehensive [16,20,21], although given the pace of publication on these topics, no article can be exhaustive. The present article is intended as a high-level argument that is informed by recent empirical trends, specifically those from studies which analyse the internal representations of neural networks. I cite only enough work as is sufficient to support the argument, trying to highlight the broad spectrum of types of studies that support the claims I make. In terms of the exposition, I will offer the most details on my own laboratory’s work, because I am most familiar with our own studies and because most of those studies were conceived of with these debates in mind.

## 2. Symbolic structure

### (a) Open questions

One of the most prominent arguments that LLMs cannot be models of language understanding in humans is that neural networks fundamentally lack the ability to encode abstract symbolic structure. This criticism applies to neural networks in general, not just LLMs. Support for this position comes from two unambiguous empirical trends. The first trend is that human behaviour, across many domains, is explained very well by traditional symbolic models, i.e. models that represent concepts as discrete constituents which are manipulated using abstract operations such as variable binding and logic [22]. The second trend is that neural networks routinely fail on exactly the phenomena where such symbolic models succeed, for example, logical inference [6,9], compositionality [7,23] and out-of-distribution generalization [24–26].

Of course, symbolic models have their weaknesses as well. Within language, for example, human inferences show high sensitivity to so-called ‘content effects’ [27] which pose challenges for the assumed role-filler independence that underlies much of compositional semantics [27–32]. Thus, despite a historic debate that has treated neural networks and symbolic systems as mutually incompatible [17] (and despite some unfortunate lingering dogma, which [33] calls out), many AI and cognitive science researchers today embrace the idea that neuro-symbolic architectures are necessary to adequately model human cognition.

Given this context, this article is not debating whether symbolic structures (in some form) are necessary to explain human cognition; I will take for granted that they are. The open question is what form these ‘symbols’ can take and where they come from. In particular, the question of interest is whether a neural network, with no inherent inductive biases, can learn to implement representations that match the explanatory power of traditional symbolic components.

Generally speaking, the popular viewpoint is that they cannot. Most hybrid neuro-symbolic systems rely on traditional symbolic components to handle the aspects of language most suited for symbolic computation, and conventional neural networks for the rest. For example, systems use neural networks to weight or re-rank candidate symbolic programs for a domain [34], or use symbolic operators to join or compose the outputs of neural perception modules [35]. Such systems are powerful and flexible, but they are not the only way in which we might instantiate a ‘middle ground’ between symbols and neural networks. An alternative approach is that the neural network implements the symbolic components implicitly, within its parameters, eliding the need for explicit symbolic components altogether.

Indeed, the in-principle potential for neural networks to implement symbolic structures is not up for debate. Even [17], in their famous criticism of neural networks, grants that they can serve as implementations of symbolic architectures, a viewpoint still held by contemporary cognitive scientists in the same tradition:

It remains open that DNNs might mimic the performance of biological perception and cognition across a wide variety of domains and tasks by implementing core features of [symbolic systems]…the competences of biological minds will require implementing a class of structured representations that uses discrete constituents to encode abstract contents and organizes them into inferentially promiscuous predicate-argument structures that can incorporate role-filler independence [22].

Thus, the primary debate is not whether neural networks can implement the relevant structures. Rather, it is whether they *do*, or more precisely, whether they can *learn to* implement them, despite their simple training objective and (at least where this debate is concerned) minimalist architectures.

A secondary debate is whether, assuming models do learn to implement these representations, the resulting objects can truly constitute symbols, as opposed to being approximations or ‘mere implementations’ of symbols ([36] summarizes some of this debate). This line of discussion is, I believe, a distraction at the present moment. If the answer to the first question is positive, then the second debate becomes an empirical question. That is, either the traditional symbolic representations and the neural implementations thereof make the same predictions about humans (i.e. about their behaviour and about other measurable properties such as neurological or processing signatures), in which case the question is moot. Or, alternatively, they make different predictions, in which case our preference between them should depend on the results of the relevant experiments.

Thus, in this section, I focus on the first question: when we inspect the internal representations of modern neural networks, do they reflect aspects of traditionally symbolic structure? Specifically, do they encode discrete constituents, organized within abstract predicate-argument structures, which combine productively? It is worth noting that the evidence below focuses primarily on a few basic aspects of the structure of the constituent concepts. The more general argument that NNs implement symbolic systems in a traditional sense [37] will depend on whether NNs *manipulate* symbols using abstract algorithms and data structures. I do not focus on this latter point only because the research has not yet been done to a sufficient extent. However, positive results concerning concept representations, of the type I do highlight, have already spurred work on characterizing the algorithms which operate over those concepts, and early evidence (discussed in §2b) suggests reason to be optimistic.

### (b) Empirical data

Large neural language models, of the type discussed in this article, have existed for roughly 5 years. Since their advent, much work has attempted to understand the internal working of these models, rather than treating them as ‘black boxes’ [38]. Emerging trends from such work suggest that internal representations are often highly reminiscent of syntactic and semantic structures assumed by traditional linguistic and cognitive science theories. Of course, even at the pace of AI research, 5 years is a short time; tools and methods for this type of analysis are under active development and results are subject to change as the work progresses. However, the important conclusions are often consistent across studies using different methods and models, and it would be misguided to dismiss these trends on the basis that the work is not fully mature.

As discussed in §1, there are indeed the countless examples of neural networks—even very large and otherwise successful ones—performing poorly in cases where conventional symbolic models would excel, e.g. cases involving compositional generalization [7], filler-role independence [6] and logical inference [9]. I do not intend to dismiss these results entirely, but rather to argue that we cannot interpret them meaningfully until we have characterized the representations and processes that underlie them.

#### (i) Compositionality

Compositionality and systematicity are central to discussions about symbolic systems. I take the relevant definition to be that given for systematicity in [17]: ‘The ability to produce/understand some sentences is intrinsically connected to the ability to produce/understand certain others…[they] must be made of the same parts’. This definition requires, for example, that in computing the meanings of the sentences *Jo loves Sam* and *Sam loves Jo*, the system employs the *same* representations of the constituents (*Jo*, *loves*, *Sam*), the *same* representations of the syntactic/semantic roles (i.e. arg1 and arg2 in *arg1 loves arg2*), and the *same* functions for combining those meanings. (cf. [39], who offers a more permissive definition which almost all modern neural networks meet by definition.)

### (c) Discrete concepts

A basic prerequisite of this definition of compositionality is the existence of discrete, modular representations of individual concepts, including representations of the roles that those concepts fill. Neural networks are known to often *entangle* concepts in a way that would violate this requirement. However, the fact that neural networks sometimes entangle concepts does not mean they always do, and a number of recent studies show that there are many cases in which neural networks learn representations which can be localized to specific parts of the parameter space and can be isolated from other (even frequently co-occurring) concepts.

**Figure 1.**
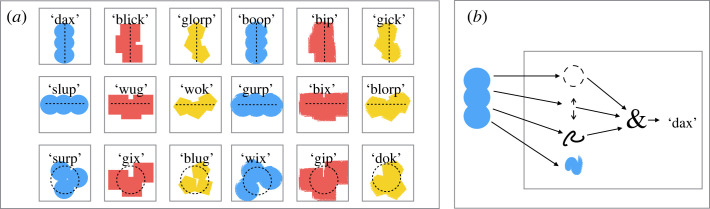
(*a*) Abstract visual classification task used in [40]. In this task, compositional concepts were defined to be combinations of shape (oval, rectangle or polygon), layout (horizontal, vertical or ring) and stroke (smooth or fuzzy), and were associated with arbitrary labels (e.g. *dax*, *wug*). (*b*) An idealized symbolic model. In this model, input observations are represented as a conjunction of discrete constituent features, which are then composed in order to determine the label.

For example, in [40], we present experiments for a variety of neural network image classification models trained on an abstract visual reasoning task (figure 1*a*). In this domain, abstract arrangements of shapes are given arbitrary names (*dax*, *blick* etc.). The data are generated such that these names depend on three underlying constituent concepts, *shape*, *layout* and *stroke*. Models trained end-to-end on the higher-level concept labelling task (i.e. with no training to explicitly encourage encoding information about shape, stroke or layout) nonetheless learn representations of the constituent concepts. These representations can be localized to linear combinations of activations at a given layer, and operate such that two key properties hold. First, the representations of the constituents are reused across different high-level concepts. That is, the representation of *circle* in the context of *dax* is the same (encoded by the same linear combination of activations) as the representation of *circle* in the context of *wix*. Second, the representations are modular. That is, it is possible to ablate the representation of the constituent shape at a given layer without impacting the model’s knowledge of the layout or the stroke. These properties are highly consistent with what we would expect from an explicitly symbolic model designed to solve the same task (figure 1*b*).

Our results are not an isolated example, but are consistent with multiple other studies that have been presented on very different models and using very different methods. For example, some work has looked for specific parameters corresponding to constituent concepts. [41] famously found evidence of a ‘cat neuron’, a phenomenon that has been remarkably replicable across newer models and analyses [14,42,43] (albeit, some studies focus on dogs, not cats, but I hope the point still stands). In language models, Geva *et al*. [44] find specific parameters which encode concepts such as statements about time, independent of their format (e.g. *between 15.00 and 19.00*, *19.00 pm Friday until…*) or expressions of part-of relationships, independent of the content (e.g. *one of the team*, *among the top ten*).

Findings of this type are not limited to first-order patterns like shapes or topics, but have also been shown for abstract categories. For example, one very consistent result in recent years has been that LLMs, especially as they increase in size, appear to capture even complex aspects of natural language syntax [16]. Evidence comes not only from performance, but from studies which suggest underlying competence as well. For example, [45] show that LLM’s embedding spaces can be decoded into explicit parse trees which achieve comparable accuracy to NLP’s best parsing models, and [46] suggests that even basic recurrent networks on formal languages can implicitly encode grammatical roles. [47,48] Show that these models encode not just parse trees, but a variety of traditional language processing components, such as parts of speech, parsing, semantic roles and coreference. Other work has found evidence for individual neurons that correspond to abstract grammatical concepts such as gender [49] and tense [50]. Taken together, such findings lend support to the argument that neural networks represent not only constituent concepts (*fillers*) but also abstract syntactic or semantic *roles*. The mere representation of roles is of course not the whole story (discussed below), but failing to find such representations would be a non-starter. Thus, at the very least, neural networks meet the basic prerequistes of our definition of compositionality.

### (d) Causality

One way to show that neural networks contain more that just the basic prerequisites is to demonstrate a *causal* relationship between the internal representation of the concepts (fillers or roles) and the behaviour of the system. Without evidence of such a causal relationship, the presence of symbol-looking representations within the neural network can be dismissed as purely epiphenomenal.

In the past few years, multiple studies have suggested that these apparently symbolic representations do indeed play a causal role in models’ subsequent predictions, each study using a different method to arrive at this conclusion. Early studies showed that it is possible to use linear [51,52] or nonlinear [53] updates to a model’s hidden state in order to manipulate the model’s representation of grammatical role, e.g. causing it to treat a noun as a verb. More recently, [54] developed a procedure for aligning individual activations of a neural network to an explicit causal graph, and used it to show that language models’ inferences obey abstract syntactic and semantic rules. [55] Presents a related study which intervenes on specific parts of the Transformer architecture [56] in order to make counterfactual edits to the model’s encoding of factual knowledge (what if *Pierre Curie’s area of work is medicine*?). They then observe that the model’s subsequent inferences (e.g. expectations about Pierre Curie’s professional accomplishments) are updated accordingly. Our own recent work has shown that it is possible to locate causal subnetworks within a model’s weights which encode concepts such as *insideness* (for vision models) or *singular nounness* (for language models), and that these subnetworks can often be turned on and off with the desired effects [57].

Methods for manipulating the internal states of neural networks are very new and thus each of these individual studies deserves scrutiny. However, the overall positive trend is consistent: not only is it possible to align neural networks’ representations *post hoc* to human interpretable concepts, but more importantly, the parts of the representation that permit this alignment are very often the same parts of the representation that the model uses to make decisions and perform tasks. This general conclusion appears robust across many very different methods, models and tasks, and holds for basic concepts (*Pierre Curie*, *insideness*) as well as for syntactic roles (part of speech, dependency structure). There is thus reason to be optimistic that, even as methods are refined and assumptions are relaxed, this higher-level trend may persist.

#### (i) Inductive biases

Compositionality is just one common argument used to favour explicitly symbolic models in cognitive science. A related but different argument is that humans are known to have many innate inductive biases that enable them to solve tasks with minimal training examples. While symbolic models can readily encode these biases, neural networks require many orders of magnitude more training data than any human can reasonably be assumed to get [58], and even after that training, they often generalize based on heuristics rather than compositional properties of a task [24–26].

Such arguments are valid, and make clear why we should not consider a randomly initialized neural network to be a good model of human language or cognition. However, pretrained language models, especially large ones, appear to have significantly improved sample efficiency when learning new tasks [1,59,60], even performing some traditionally symbolic tasks well with only a few training examples [61]. Our own work has suggested that LLMs' preference for one solution over another is a function of the description length of the candidate solutions and, moreover, that pretraining often serves to lower the description length of the ‘right’ (i.e. compositional) solutions [62]. This pattern is highly characteristic of more traditional symbolic systems, which also prefer solutions with low description length [63] and have been engineered specifically to ‘refactor’ their representations during training to decrease description length of more general concepts [64]. [65] go even further in arguing that in-context learning—the primary mechanism via which LLMs exhibit sample efficiency—can be understood as an implicit implementation of more familiar Bayesian inference. Across all these studies, the fact that LLMs might obey similar principles during their training to the principles obeyed by probabilistic symbolic systems provides evidence that the processes they use under the hood may reflect those that traditional theories in cognitive science routinely employ.

As ever, skepticism about the sample efficiency of LLMs is warranted. Models of this size are often proprietary, and even when the data is publicly available, determining whether a task or concept is ‘unseen’ is not trivial [66]. These claims demand further study. Still, based on what we know right now, pretrained LLMs are not obviously incompatible with human-like sample efficiency, and in fact may be governed by similar constraints as competitive symbolic models in similar settings.

### (e) Summary and discussion

A common criticism of neural networks as candidate models of the human mind is that neural networks lack the abstract symbolic structures and processing algorithms necessary to explain human language. Such claims are typically supported by evidence of neural network performance failures on tasks that traditionally require symbolic processing. I argue that if we focus instead on research which seeks to assess the underlying competence of neural network models—i.e. work which characterizes the structure of the representations that models use under the hood—the picture is more positive, with neural networks reflecting many characteristic aspects of symbolic systems.

Of course, there are many important aspects of symbolic processing to which the evidence presented in the above sections does not speak. Logical inference is one obvious example. Variable binding is another. Such properties are central to symbolic models, and are a primary reason why many cognitive scientists still favour such models as explanations of human behaviour. If, as discussed in §2a, the ultimate question is whether neural networks can match the explanatory power of symbolic models across these varying domains, we need to find evidence that the neural networks possess the underlying competence to perform (something at least resembling) these aspects of symbolic computation. At the moment, we do not have this.

However, a primary reason is that we have only just started to look. At least at this early stage, the absence of evidence should not be interpreted as evidence of absence. The methods described above for uncovering representations of symbolic concepts and even abstract roles do not readily generalize to discovering representations of functions (as is needed for these next steps). However, other methods will likely surface to bridge this gap. For example, a close investigation of the Transformer architecture by Olsson *et al*. [67] suggests that the model’s attention heads interact to implement abstract memory reads and writes. [44] show that Transformers’ feed-forward networks appear to implement abstract key-value stores. These findings might foreshadow the implementation of more abstract symbol manipulation. Moreover, work which analyses the generations of language models finds that they are capable of generating grammatical but unattested syntactic structures [68], a finding which strongly suggests that some mechanism for abstract variable binding must exist, even if we have not yet characterized it.

Thus, while the unanswered questions far outnumber the answered ones, at this early a stage in the investigation, there is reason to be optimistic. The next decade of work is poised to uncover neural network implementations of at least some non-trivial competencies traditionally seen only in explicit symbolic systems. Even if they fall short of capturing everything we need to explain cognition, such findings could still significantly inform how we think about representation of these competencies in humans.

## 3. Grounding

### (a) Open questions

The above-section discussed a common criticism of neural networks in general. However, LLMs in particular are often criticized not because they are neural networks, but because they are trained only on text, via a very simple next word prediction objective. As such, they have no access to or awareness of the ‘real world’ to which language refers. Many have cited this property as the reason why LLMs, by definition, cannot encode what is standardly referred of as ‘meaning’ and thus cannot be models of human language in any deep sense. For example:

[T]he language modeling task, because it only uses form [text] as training data, cannot in principle lead to learning of meaning…We take (linguistic) meaning to be the relation between a linguistic form and communicative intent…When humans use language, we do so for a purpose: We do not talk for the joy of moving our articulators, but in order to achieve some communicative intent [69].

GPT-3 has no idea how the world works…The immense database of things that GPT draws on consists entirely of language uttered by humans, in the real world with utterances that (generally) grounded in the real world. That means, for example, that the entities…and properties…generally refer to real entities and properties in the world …[but] GPT doesn’t actually *know* which of the elements appropriately combine with which other properties [70].

Such arguments are extremely compelling because they are based on a premise that is impossible to disagree with. When humans learn and use language, they are embedded in a rich non-linguistic world, interacting via perception, communication, planning and goals. Text-only language models are not. However, the face-validity of the premise hides the subtlety of what is at-issue in the conclusion. In fact, there is not consensus among philosophers and cognitive scientists about the extent to which grounding is a key component of what we conventionally refer to when we use the word ‘meaning’.

Here, it is worth differentiating claims about the need for communicative intent from claims about the need for an external world more generally. (Perception is the most obvious example of the latter category, but there are other examples, e.g. an agent’s non-communicative goals.) Arguments against LLMs often entangle both into a generic notion of grounding, but these claims are not the same. They inherit from separate philosophical traditions and thus have separate sets of criticisms to address. (cf. [71,72] who present partially overlapping arguments with the ones I make below.)

#### (i) Does meaning require communicative intent?

A common line of argument is that LLMs cannot encode meaning because they lack communicative intent. That is, when humans *mean* things by language, it is by virtue of the fact that they are using language to influence others’ thoughts and actions. This notion of meaning originates from [73] who argues that meaning is dependent on the way in which words are used. This perspective is appealing to natural language processing researchers because it is (one of) the possible theoretical traditions that is consistent with the distributional hypothesis [74,75] on which nearly all modern computational models of language are based.

However, the claim that the meaning of a symbol is *defined by* (i.e. as opposed to merely related to) its role in communication is not universally endorsed. The competing view argues that symbols derive much, or even all, of their core meaning via their role in *internal computations*. [76] Summarizes this distinction:

[It is useful to distinguish] (at least) two uses of symbols, their use in calculation, as in adding a column of figures, and their use in communication, as in telling someone the result. Symbols that are used in calculation are typically not being used at the time for communication…You might invent a special notation in order to work out a certain sort of problem. It would be quite proper to say that by a given symbol you mean so-and-so, even though you have no intentions to use these symbols in any sort of communication [76].

The above position, that meaning is in large part a product of internal (to the mind) computation is not fringe. In fact, it is arguably the dominant view among modern linguists and cognitive scientists. That is not to say modern linguists and cognitive scientists deny that spoken and written language is communicative; it obviously is. Rather, the point is that cognition (including thinking, planning, etc) is often best modelled in terms of manipulations of meaningful symbols, and these symbols have meaning whether or not they are attached to communication. According to this position, the debate about whether an LLM can encode meaning does not depend on whether it has communicative intent when it predicts the next word in a sentence, but rather depends on the form of the internal computations that connect its inputs to its outputs.

Notably, the debate about whether meaning derives from communication versus computation does not reduce to the debate between formal versus distributional semantics. It is true that formal linguistics and associated philosophy takes meaning to be computation [77,78], but that is not the feature that differentiates formal from distributional semantics. Even within theories that endorse the slogan ‘meaning in use’, it is still necessary to distinguish whether the relevant ‘use’ is communication or computation. As mentioned, a Gricean tradition might take the relevant ‘use’ to be communication, but an equally natural philosophical home for modern natural language processing models is conceptual role semantics (CRS), which is ‘a version of the theory that meaning is use, where the basic use of symbols is taken to be in calculation not in communication’ [76]. Under CRS, the meaning of a symbol (word) is a function of the contexts in which it occurs, where communication is just one (possibly small) part of this context. Within CRS, it is possible to argue, for example, that the role of a symbol in communication has a negligible effect on its meaning compared to the much more substantive effect of e.g. the role of the symbol in inference.

#### (ii) Does meaning require (other forms of) grounding?

Even if one accepts, via something like the above argument, that meaning does not require communicative intent, it is possible to still maintain a need for grounding to something other than communicative intent. For example, non-communicative meaning might still require grounding to perception (*red*), to cognitive or emotional states (*furious*), to biological needs (*hungry*), or to abstract goals (*get*), among other things. In response to such claims, there are multiple lines of possible argument. Below, I summarize two.

The first appeals to the same theory, CRS [76,79], described above. Under this account, the meaning of a symbol is derived (mostly or entirely) from its relation to other symbols, i.e. its role in the thoughts and inferences in which it participates. The ability to refer may be part of its role, but it need not be the primary part. For example, we are happy to use noun phrases like *the first baby born on 1st July 2037*, which acquire their meaning via definition, not by virtue of referring to any real entity. These arguments are not even limited to abstract or hypothetical concepts. [80] studied children’s understanding of the concept *raccoon* and revealed that no perceptual, functional, or in any way observable properties appeared to alter beliefs about what makes a raccoon a raccoon, leading to the conclusion that the meaning of *raccoon* is some unobservable ‘essence’. [81] Thus argue that many such common concepts are embedded in theories and that their meaning is derived from the role they play in those theories. Within CRS, one can argue that in fact *all* words acquire their meaning in the same way. Under such a theory, the question of whether LLMs encode meaning depends on the structure of the concepts they encode internally, and the relationship between those concepts.

The above argument might be considered an *internalist* account, in which meaning depends only on the structure of the internal representations. However, even if we are seeking an *externalist* account of meaning, there is a line of argument via which to defend LLMs. Specifically, for many (most!) concepts that we ‘know the meaning of’, we do not observe their referents directly. Rather, the words retain their referential power via a causal chain [82] linking the symbol to the external world. Consider, for example, the meaning of *Jean Pavlick* (my paternal grandmother). Almost certainly no one reading this has met, or even seen a picture of, my grandmother. You are learning the meaning of the phrase *Jean Pavlick* just now. Still, this does not make the word meaningless, or even reference-less. Rather, you inherit the grounded meaning from me, who has directly observed the referent (she’s lovely). Thus, for a very large number of concepts, we retain a grounded meaning purely by virtue of learning the meaning from someone who exists along a causal chain connecting the symbol to its referent. Essentially: we know a guy who knows a guy who knows a guy who has directly observed the referent. Chater [83] echoes this argument (ironically, in the context of arguing *for* a need for communicative intent):

Human intelligence is inherently collective: the creation of language, conventions, norms, systems of religious and scientific belief, technologies and institutions and organisations of all kinds are far beyond the powers of any individual human mind. Rather, we are like termites each making only a small local contribution to a vast edifice whose extent and complexity far exceeds our understanding [83].

Adopting this collective view of human intelligence, language models reading text on the internet, which was written by a human, are as good a member of the causal chain as anyone. A language model learning about *red*, or *hilarious*, or *soft*, never having seen, experienced, or felt those things, is no different than you or I learning most of what we know. Indeed, [84] put forth such an argument, along with extensive empirical support, to illustrate how congenitally blind children are able to develop language largely in line with their sighted peers.

#### (iii) Takeaway

It is not important for the purposes of this paper which of the above theories or arguments is correct. What is important is that the debate is open, and informed people can disagree about the role that grounding plays in humans’ meaning representations. Thus, if we can grant that the lack of grounding does not automatically disqualify LLMs from being models of the mind, the question becomes about whether or not LLMs are demonstrably bad at explaining human behaviour, in particular concerning the types of concepts where we might expect grounding to be an important factor.

### (b) Empirical data

Recent work has sought to determine whether there exist meaningful, measurable differences between the (ungrounded) conceptual representations encoded by LLMs and those encoded by a grounded model of the same concepts. If there are no measurable differences between the representations, then there is little basis to claim that grounding matters for the questions at hand. By contrast, if there are differences, then they cannot both be equally good models of the mind, and new human data should eventually adjudicate between them.

There are currently two approaches researchers have taken to attempt to address this question. The first focuses on language-only tasks (i.e. tasks in which the system receives only text as input and can produce only text as output) and seeks evidence that the performance of text-only LLMs differs from the performance of a grounded system. The second approach focuses on the geometry of the conceptual spaces themselves, and seeks to show that the representations within LLMs are structurally equivalent to the representations of a grounded model. I will focus primarily on experiments of this second type. Studies of the first type have methodological limitations which make their conclusions difficult to interpret at this time. Thus I will comment on this line of work only briefly.

#### (i) Mapping between grounded and ungrounded spaces

One way to show that grounding is not necessary for learning meaning is to show that conceptual representations learned by an (ungrounded) LLM are *isomorphic* to grounded representations of those same concepts. That is, we want evidence that there exists a mapping between the spaces that preserves all the individual concepts and the relations between them. If such a mapping exists, then any computation carried out in the grounded space could in principle be carried out in the ungrounded space and yield the same result, in which case the spaces might as well be the same. (Of course, further work would be required to show that indeed there is an equivalence between the computations carried out by grounded versus ungrounded agents. But showing an equivalence between the representations is a necessary first step in this direction.)

As of writing, there is no formal proof, to my knowledge, that bears directly on this argument. [85] propose a proof that no isomorphism can exist between an ungrounded LM and a formal semantic representation of meaning. However, the conclusions of the proof (that the spaces cannot be isomorphic) depends on an assumption about meaning as reference which is not compatible with contemporary models of meaning in humans. Thus, relevant insight on this question comes primarily from empirical studies.

For example, in [86], we investigated the ability of LLMs to map terms for colours (*red*, *navy*) and spatial directions (*right*, *northwest*) to a grounded representation on the basis of a small number of examples. We can make an analogy here to being lost in the woods, and the fact that a person, being shown which direction is north and east, can immediately infer south and west. Similarly, for an LLM with a correct (but not yet grounded) representation of these concepts, a small number of well-chosen examples should be sufficient to infer the grounded meaning of the entire space. We found that the largest LLMs are able to perform such grounding significantly above chance (albeit far from perfectly). Moreover, the LLMs are able to generalize the mapping to unseen subspaces. For example, being shown examples of only the primary and secondary colours, plus 54 shades of pinks and reds (*crimson*, *brick*, *salmon* etc.), the LLM is able to infer the correct word for distant colours such as *navy*.

Such findings are largely consistent with earlier work by Abdou *et al*. [87], which looked specifically at the mappability of LLM representations for colour words to the perceptual similarity of those colours (using CIELAB encodings). [87]’s conclusions were generally positive, reporting similarity between the spaces, but with some caveats stemming from colour words with multiple senses (e.g. *orange*, *violet*). However, given the CRS theories discussed above, the mixed negative results in [87] are a feature rather than a bug. That is, LLM representations of colour words *should* be influenced by aspects conceptual role other than perception. Thus, taken together, [86,87]’s results suggest a positive finding that LLMs can behave as though they are aware of perceptual features associated with words, but encode richer aspects of conceptual role as well.

**Figure 2.**
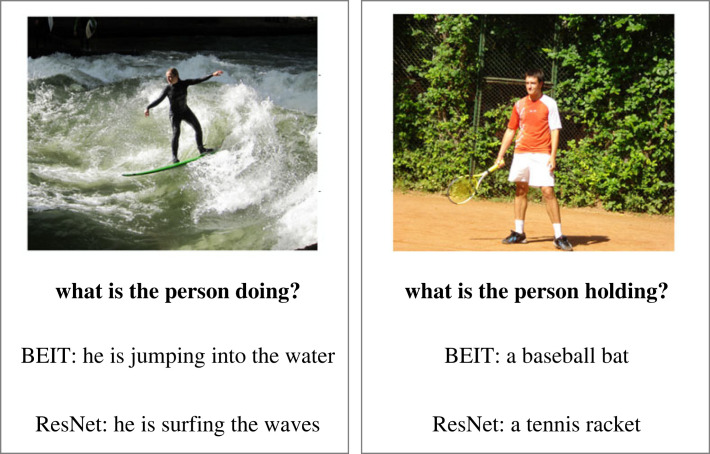
Example from [89], in which a (ungrounded) language model is used to generate captions for images by linearly projected the image encodings into the language model’s input space. The examples above show captions generated when using two different image encoders: ResNet, in which the image encoding is trained on an image classification task and thus implicitly has access to linguistic supervision via the linguistic categories (e.g. animal names); and BEIT, in which the image encoder is trained on a reconstruction objective with no access to linguistic signals.

Work that aims to map between LLM and perceptual space has not been limited to narrow domains like colour. [88] and more recently [89] investigate whether it is possible to project entire images into an LLM’s representation space, for example, to generate a description, or to ask or answer questions about the image. While other work has done this using various deep learning architectures [88–91] are notable for using only a simple linear projection in order to perform this mapping. Despite the simplicity, both papers report positive results. For example, in [89], we found that an LLM which was trained only on text could generate captions with a surprising level of detail if the image was encoded by a multimodal system (i.e. one with access to both language and vision during training). Perhaps more impressively, even when given an image encoded by a system with no linguistic knowledge whatsoever, the LLM was able to generate a caption with course-grained conceptual awareness (e.g. mislabelling a tennis racket as a baseball bat) (figure 2).

#### (ii) Measuring grounding using text-only tasks

Work which directly seeks to find mappings between grounded and ungrounded spaces is comparatively less common than work which analyses models in text-only settings. For example, several studies design question answering datasets such that answering the question correctly purportedly requires reasoning about the physical world (e.g. colour, shape, motion) [10,11]. In order to prevent models from succeeding using heuristics, these datasets generally involve reasoning about highly improbably scenarios, e.g. *Get your sofa onto the roof of your house, without using a pulley, a ladder, a crane* [11]. Such studies reveal that LLMs perform very poorly compared to humans, and are thus taken to be evidence that the LLM requires grounding in the physical world in order to succeed. A separate approach uses ‘probing classifiers’ [92,93] in order to measure the extent to which internal states of the model encode information about physical properties. For example, [94] look at whether word embeddings in an LLM encode information about objects’ physical attributes and affordances. [95] perform similar analyses. These studies have tended to be more positive than those which are based on question answering tasks, often reporting results that suggest the LLMs perform well above baseline, though still below humans.

Studies like these, in which text-only LLMs are evaluated in text-only settings, help clarify the current state of what LLMs can and cannot do, but it is hard to interpret their results—either positive or negative—relative to the questions under discussion in this paper. One reason (relevant for the studies that rely on question-answering, but less so for the probing studies) is the performance-competence argument discussed in §1. But, specifically in the context of the grounding question, there is a second reason these studies are difficult to interpret. Namely, they usually compare the performance of an LLM to human performance, but do not report performance of any grounded model. Thus, to the extent that the LLMs underperform humans (which is always the case), we cannot conclude whether a lack of grounding is the reason for the gap. This limitation is not sloppiness on the part of the authors—running such a comparison is difficult if not impossible given current technology and resources. It would require two models which are trained on identical language data, one with access to ‘grounding’ (which could be instantiated in countless possible ways) and one without. And training such models assumes we know the right way to give the model ‘grounding’, which we certainly do not. In fact, we attempted to run such a study in [96]. We reported a null result (i.e. no measurable difference between the grounded and ungrounded models), but the result was unsatisfying for exactly the reasons just stated. In order to train comparable models, we were limited to language from very restricted domains (cooking videos). Even setting aside the issue of data, the null result could always be due to our having trained the model badly, e.g. choosing a bad method for integrating modalities. Thus, even in this context in which I am actively arguing that grounding is not required for meaning, I would not point to our results from [96] as evidence in favour of the claim. Simply put, more work is needed before we can draw strong conclusions, especially given negative results, from studies of this type.

### (c) Summary and discussion

LLMs in particular are frequently dismissed as candidate models of linguistic meaning due to their lack of grounding—i.e. the communicative, perceptual, or goal-oriented contexts in which language occurs. However, in cognitive science and philosophy of language, there is disagreement about the extent to which grounding constitutes an essential component of what is commonly referred to as ‘meaning’. As such, there is a legitimate theoretical basis for claims that LLMs, even without explicit grounding, can nonetheless be said to encode ‘meaning’. Moreover, recent empirical work has suggested that it is often possible to align the conceptual space of LLMs to a conventionally grounded space, even enabling LLMs to learn to perform tasks such as image captioning with no changes to their internal representations. While such results are new and underexplored, they suggest an avenue via which we could treat LLMs as legitimate models of humans’ mental representations despite the overt disconnect between humans’ and LLMs’ learning processes.

Assuming work in this direction is pursued, the questions discussed in this section necessarily intersect with those discussed in §2. That is, important questions about how LLMs implement (grounded or ungrounded aspects of) conceptual role will depend on questions about the structure (symbolic or otherwise) of those representations and the causal connections between them. These questions are paramount to our understanding of meaning in language and cognition, and research in this direction could substantially influence the as yet philosophical debates on the issue.

Of course, there are many questions beyond only whether LLMs can serve as models of meaning. As [83] argues, ‘intelligence is inherently social, and artificial systems, like humans, will be viewed as intelligent, and will be valued as collaborators, to the extent that they can align and coordinate their thoughts and actions with human thoughts and actions’. It may well be the case that showing that LLMs exhibit *intelligence* is a higher bar than showing they encode meaning, and it may be that communicative intent and other types of grounding will play a crucial role in such debates. However, LLMs' failure to account for all of human cognition (e.g. full-blown intelligence) does not prevent them from serving as useful models of some aspects (meaning). And, in fact, seriously investigating the potential of LLMs to shed light on the former may incidentally generate insights about the latter.

## 4. Conclusion

It is an open question whether the success of LLMs can offer insight on the study of language understanding in humans. Two common arguments against LLMs as models of humans are (i) the fact that LLMs lack the capacity to represent abstract symbolic structure and (ii) the fact that LLMs are trained only on text and thus lack grounding. Support for both claims is typically based on either in-principle arguments, or else on evidence of LLMs performing poorly on tasks that require symbols or grounding, respectively.

In this article, I argued that neither criticism of LLMs can be accepted *a priori*, and rather, both claims must be tested empirically. In particular, for those interested in the potential of LLMs to model cognition, the priority must be on characterizing the models’ underlying *competence*, rather than focusing on measures of their *performance* (good or bad). Recent empirical work on the former gives reason to believe that neural networks can learn to encode many key aspects of traditional symbolic structures, and that even ungrounded language models can encode a conceptual space that is structurally similar to a grounded space. Overall, I conclude it is premature to make claims about intrinsic (in)abilities of LLM. Rather, the next decade of empirical work is likely to significantly influence our understanding of the relationship between artificial and human language understanding.

## Data Availability

This article has no additional data.

## Footnotes

1 My use of the words *performance* and *competence* is a deliberate re-imaging of Chomsky’s terminology. Chomsky argued that the presence of language production errors in humans is not evidence against an internal representation of some abstract, ideal grammatical structure. Similarly, the fact that we readily find examples of LLMs producing illogical or inconsistent outputs should not alone be taken as evidence that they do not represent some more abstract or robust structure internally. If we focus on understanding this internal structure first, we might notice connections between processing in LLMs and processing in humans which would be missed if we focused solely on LLMs' errors in performance.
